# Perinatal psychological interventions to promote breastfeeding: a narrative review

**DOI:** 10.1186/s13006-020-00348-y

**Published:** 2021-01-06

**Authors:** Lidia Gómez, Sergio Verd, Gloria de-la-Banda, Esther Cardo, Mateu Servera, Ana Filgueira, Jaume Ponce-Taylor, Margarita Mulet

**Affiliations:** 1 Department of Child Psychiatry, Son Espases Hospital, Valldemossa road, 07120 Palma de Mallorca, Spain; 2 Baleares Medical Research Council (IdISBa), Valldemossa road, 07120 Palma de Mallorca, Spain; 3 Pediatric Unit, La Vileta Surgery, Department of Primary Care, Matamusinos street, 07013 Palma de Mallorca, Spain; 4 Department of Psychology, Baleares Islands University, Valldemossa road, 07122 Palma de Mallorca, Spain; 5 Department of Paediatrics, Hospital Son Llatzer, Manacor road, 07128 Palma de Mallorca, Spain; 6 Accidents & Emergency Unit, Department of Primary Care, Illes Balears street., 07014 Palma de Mallorca, Spain; 7 Mental Health Unit, Department of Primary Care, Simo Tort street, 07500 Mallorca, Manacor Spain

**Keywords:** Breastfeeding, Postpartum, Psychotherapy, Pregnancy, Depression, Narrative review, Anxiety, Perinatal

## Abstract

**Background:**

Emotional distress in mothers inhibits the let-down reflex, thus affecting breastfeeding self-efficacy. A breastfeeding mother may have to cope with both physical discomfort and psychological distress. However, literature on initiatives to improve breastfeeding rates has focused mainly on providing community-based peer support, or social policies. The aim of this review is to assess evidence on the effectiveness of a broad range of psychological interventions to facilitate breastfeeding for mothers facing difficulties around the time of delivery.

**Methods:**

The review of the literature is derived from a search on Cochrane Library, PubMed, EBSCOhost, and PsycINFO for papers published since 1980. The approach was to explore quantitative and qualitative parameters. Quantitative parameters included breastfeeding initiation, duration, and composition. Qualitative parameters recorded the evaluation of maternal perceptions on breastfeeding success. The high heterogeneity of the studies led to a narrative review; 20 selected papers that report on breastfeeding outcomes and psychological programs met the inclusion criteria.

**Results:**

The evidence on breastfeeding support through psychotherapy is heterogeneous and scant. Out of the included studies, 11 were randomized controlled trials, two were non-randomised trials, and two used a quasi-experimental design. None of the studies reported an increase in adverse breastfeeding outcomes. Three studies failed to report an association between psychological procedures and improved breastfeeding outcomes. A literature review showed that 17 (85%) analyses support stress-releasing techniques to facilitate breastfeeding.

**Conclusions:**

This review suggests that relaxation interventions carefully tailored to address perinatal emotional distress may lead to important health benefits, including improvement in breastfeeding outcomes. There is also some indication that psychotherapy support while breastfeeding may have more impact than routine counselling. Conversely, this review did not find an association between self-hypnosis and breastfeeding outcomes. Data from this study can be used in designing prevention programs and future research with appropriate theoretical underpinning.

## Background

Medical research continually reinforces the health benefits of human milk for infants. What is novel, however, is a growing body of evidence that shows how important it is to breastfeed in order to maintain good physical and mental health of recent mothers [1, 2]. Much of the debate on interventions to promote successful breastfeeding has focused on milk expression methods, peer community-based support, professional education, maternity care practices, leave policies, workplace regulations, or social marketing [3–5]. Overall, public health interventions have been effective in increasing the proportion of breastfeeding initiation [6, 7]. However, more efficient resources are essential to achieve an improvement in breastfeeding exclusivity and maintenance [8] among mothers who need extra support.

In 1858, Marcé published the first paper devoted entirely to puerperal mental illness. He reported that 33% of women developed depression in the lactational period [9]. Current research confirms that up to 5.5% of women use antidepressant drugs during the perinatal period [10]. The proportion of pregnant or lactating women that are prescribed antidepressants varies by geographical location. Across Europe, there are marked differences in the prescription of antidepressants to women during and after pregnancy, from 3.3% in Italy, to 9.6% in Wales. On the other hand, prescription of antidepressants during pregnancy is lower in Europe than in the USA, with reported percentages between 5.6 and 10.2%. In all databases, antidepressant use was at its lowest during the second and third trimesters of pregnancy, but by six months post-pregnancy, the rate of antidepressant use had returned to pre-pregnancy levels [11]. It is worth emphasizing that systematic reviews fail to draw any clear conclusions about the effectiveness of antidepressants for the prevention of postnatal depression [12]. No less important is that antidepressant prescription independently predicts exclusive formula feeding shortly after birth [13].

It is widely accepted that perinatal mood disorders (PMD) affect an estimated 20% of breastfeeding mothers [14]. Additionally, there is strong evidence for an association between higher levels of maternal depressive symptomatology and shorter breastfeeding duration [15–23]. Even when cessation of breastfeeding is due to inherited metabolic disorders, mothers who are obliged to replace breastfeeding with special infant formulas experience the highest degrees of stress [24]. Another issue under consideration, less obvious but no less important, is to what extent breastfeeding prevents mood disorders. It has been shown that the rising levels of inflammation markers during the third trimester of gestation constitute a risk factor for PMD [25, 26]. Breastfeeding may intervene to counterbalance this situation via down-regulation of both stress and inflammatory response systems [27–30].

Notwithstanding the above, breastfeeding support through psychotherapy interventions has been scarcely explored. The aim of the present study is to critically assess the effectiveness of psychological programmes (i.e. psychotherapy, relaxation, and stress-releasing techniques) on the breastfeeding success of recent mothers. Early references unveiled a multifaceted relationship between stress and breastfeeding. Severe stress may result in a negative effect on the process of lactation, both behaviourally and biologically [31, 32]. At its best, psychological support might relaunch a virtuous cycle involving the prophylactic role of breastfeeding in reducing maternal psychological distress.

## Method

### Data sources

An extensive literature search was performed up to the week starting January 15th, 2020. The primary endpoint was the assessment of breastfeeding success in mothers participating in psychotherapeutic interventions aimed to provide support to participants in enduring the difficulties associated with childbirth. For the purpose of this review, psychological therapy interventions include a wide range of interventions that target cognition, motivation and behaviour. The approach was to explore quantitative and qualitative parameters. It should be noted that this is a narrative review rather than a systematic review. Quantitative parameters included breastfeeding initiation, duration, and composition. Qualitative parameters recorded the evaluation of maternal perceptions on breastfeeding success. Databases searched were: Cochrane Library, PubMed, EBSCOhost, and PsycINFO for papers published since 1980.

Table 1 outlines the search strategies and key terms used. During the search, keywords for breastfeeding and for psychological interventions were considered primary, and were either combined to each of the other keywords individually or used in various combinations.

**Table 1:** Search terms used to identify existing literature reporting psychological interventions to support breastfeeding, 15 January 2020

No. Search strategy	Map term to subject heading (MeSH)	Keywords
1. MeSH or keywords (key findings for breastfeeding)	Breastfeeding, lactating, lactation, human milk, breast milk, breastfeed, and breastfed	Breastfeeding or “breast feeding” or breastfed or lactation or “breast milk” or “human milk”
2. MeSH or keywords (key findings for psychological interventions)	Relaxation therapy, relaxation techniques, meditation, imagery, verbal protocol, guided imagery, music therapy, cognitive therapy, perinatal mental health, psychotherapy, psychodynamics, hypnosis, cognitive behavioural therapy, interpersonal psychotherapy, group psychotherapy, imagery psychotherapy	“Relaxation therapy” or meditation or “guided imagery” or “music therapy” or “verbal protocol” or “cognitive therapy” or “perinatal mental health” or psychotherapy or psychodynamics
3. 1 and 2 (combination both of key findings)	(Breastfeeding, lactating, lactation, human milk, breast milk, breastfeed, and breastfed) or [(breastfeeding or “breast feeding” or breastfed) or lactation or “breast milk” or “human milk”] and (relaxation therapy, relaxation techniques, meditation, imagery, verbal protocol, guided imagery music therapy, cognitive therapy, perinatal mental health, psychotherapy, psychodynamics, hypnosis, cognitive behavioural therapy, interpersonal psychotherapy, group psychotherapy, imagery psychotherapy) or (“relaxation therapy” or meditation or “guided imagery” or “music therapy” or “verbal protocol” “or “cognitive therapy” or “perinatal mental health” or psychotherapy or psychodynamics)	

**Fig. 1:**
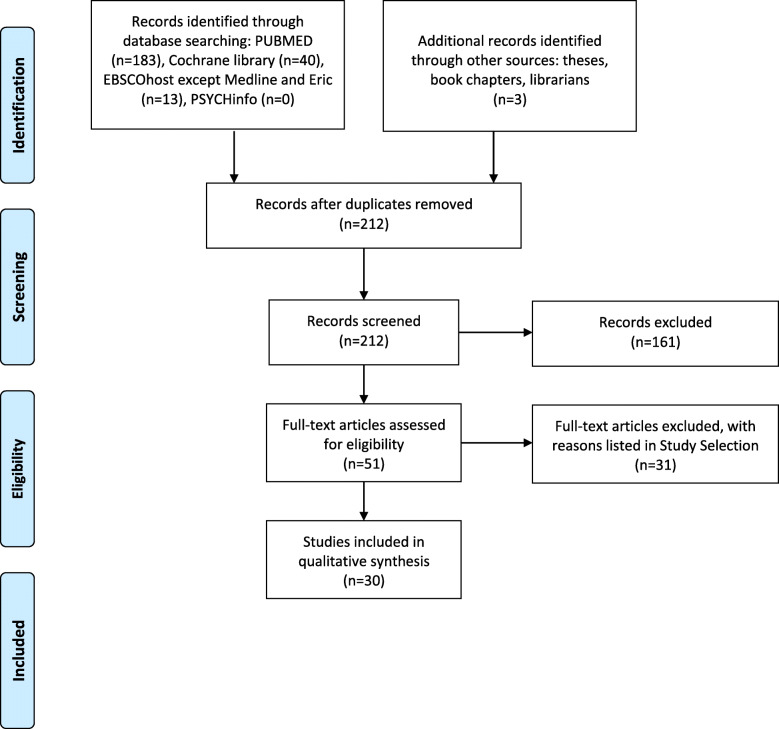
PRISMA flow diagram 1c

The search yielded 239 articles, out of which 20 articles were considered relevant for inclusion in this review (Fig. 1). First, titles and abstracts of articles from databases were screened and identified for eligibility. Selected articles were evaluated independently by two reviewers. When supporting data were not available, recommendations were made based upon the combined opinions of more than two authors. We recognize that inconsistencies can certainly occur during the searching stage. We describe what was done with the literature once it was identified, in order to assess and bias: selected studies met the following four conditions: (1) to include trials that do not substantially overlap, the main outcome of searching more than one database is that differences in indexing across databases increase the chances of retrieving relevant papers; (2) to have matching scopes, even though literature review is a cascading process of summing up materials about a topic in order to fully match the scope and innovation in a specific field; (3) to have good methodological quality; and (4) were published from 1989 to 2019 [33].

Although the hierarchy of evidence favours quantitative methods, mixed research approaches are becoming increasingly acceptable in assembling evidence. There is a place for rigorous qualitative research or case series that include in-depth analyses of few patients in their natural clinical setting.

### Study selection

Studies were eligible if they collected data relating to breastfeeding behaviour, outcomes or biological sequelae (ie. breast milk composition), and focused on psychological interventions in the *peripartum* (beginning sometime during the final month of pregnancy through about 5 months *postpartum*) [34]. Given the high variability in breastfeeding definitions, and for the purpose of this review, breastfeeding is defined as the provision of breastmilk to an infant by any means. Studies of mothers with premature or very low birthweight infants were also deemed eligible.

Conversely, studies of infants with cleft palate, cerebral palsy, gastrointestinal disorders, food allergy or other specific medical problems known to affect feeding were excluded due to the high risk of confounding. Additional exclusion criteria for this review were protocols, reviews, commentaries, and letters to the Editor.

We divided all studies included in four different sections: (1) randomized controlled trials; (2) quasi-experimental trials; (3) pre-post design studies; and (4) case series that reported more than one case report of psychological interventions to support breastfeeding. We only included single-center study data.

### Pre-registration

This study is pre-registered. A PDF document containing the AsPredicted Protocol *‘Perinatal psychological interventions to promote breastfeeding’ (#40903)* is publicly available [35]. Question 1 of the AsPredicted document shows that some data had been already collected at the time of pre-registration. In this case, preregistration involves specifying the study design, and takes place before the data are analysed.

## Results

### Study characteristics

Studies included were published between 1989 and 2019. Samples were derived from 11 countries (Australia, Canada, China, Croatia, Denmark, India, Iran, Malaysia, Pakistan, United Kingdom, and the United States). Twenty studies were finally included in the present critical analysis and are summarized here. Each study is briefly commented on, highlighting aspects of study design and results. Out of the included studies, 11 were randomized controlled trials (RCT), four were quasi-experimental, three were case-series reports, and the remaining two studies used a pre-post design. The total number of mothers included was 3136. Interventions were analysed, regardless of whether these programs were initiated antenatally or postnatally. The studies were clustered in four groups composed of two to three comparable psychological interventions.

Nine studies of the 11 included RCTs, revealed an association between breastfeeding and psychological interventions on maternal mood. On the other hand, the results of a RCT on interpersonal psychotherapy [36] and another on self-hypnosis [37] did not suggest that mothers on therapy may be more likely to breastfeed. Out of four quasi-experimental studies, the relationship between breastfeeding and mothers’ relaxation interventions was demonstrated in two studies with a pre-test-post-test design, another did not report an association between psychological therapy and breastfeeding outcomes, and another could not be merited because measurements do not apply to it. The rest were case series.

In six studies, the control group received no extra attention other than the usual perinatal care. Five of those studies reported significant differences between control and intervention groups. Conversely, in nine studies, either mothers were randomly allocated to intervention or control sessions, or mothers in experimental and control groups received the same breastfeeding support and guidance during the trial. Both groups reported equal satisfaction levels with regards to health care offered to protect breastfeeding. Out of nine studies, eight reported significant differences between experimental and control groups.

Most papers deal with universal interventions targeting a population not at increased risk for perinatal mental health conditions; only two articles reviewed are devoted to selective psychological interventions for women perceived to be at risk of perinatal depression [36, 38].

Two main different psychotherapeutic approaches have been evaluated. On the one hand, approximately half of the papers have evaluated the effectiveness of psychotherapies that are performed by psychiatrists and psychologists [36, 39, 40], and to a lesser extent by nurse therapists, social workers, or specialised counsellors under close supervision [37, 38, 41–43] (i.e. cognitive-behavioural therapy, interpersonal psychotherapy, group therapy, or hypnosis). In the remaining studies, a reference is made to research projects whose staff has received brief training in applying methods that have already been tested, and are suitable for healthcare or prevention [44–55] (manual relaxation, meditation relaxation techniques, music therapy or compassion-focused therapy).

Results are similar for interventions delivered by specialised versus non-specialised professionals. Psychological interventions delivered by specialists achieved significant results in seven out of 10 cases, and also in six out of seven interventions by non-specialists.

Study descriptions have been tabulated (Table 2), along with method quality and risk of bias (Table 3).

**Table 2:** Studies’ description. Characteristics of women and infants for whom a psychological intervention to support breastfeeding has been tested. Results reported according to study design

Study design, sample size, range or infant age	Author, country, Year, Reference	Participants at enrolment	Intervention	Breastfeeding outcomes.	Professional or self-report assessment	OR or test values or mean (S:D.); ***P*** value
RCT, *N* = 71, 1–42 DOL	Feher, USA, 1989 [54]	Mothers of preterm infants	Relaxation therapy	Breast milk yield and composition.	Professional	90.1 mL(60.0) vs 55.4 mL (48.2); *p* < 0.05
RCT, *N* = 100, 1–180 DOL	Vidas, Croatia, 2011 [40]	Nursing mothers who were ≤ 2 months post-partum	Relaxation therapy	EBF > 6 months.	Self-report	Risk ratio 11; 95% CI (3.60, 33.54); *P* = 0.0001
RCT, *N* = 162, 1–14 DOL	Keith, USA, 2012 [50]	Mothers of preterm infants	Music therapy coupled with visual reminder of the mother’s baby	Breast milk yield (DOL 14) and fat content. (DOL 6)	Professional	1028 mL (48.8) vs 591.4 mL (47.6); *P* < 0.001 60.7% (3.5) vs 50.7% (3.4); *P* = 0.001
RCT, *N* = 145	Spinelli, USA, 2013 [36]	12–33 weeks gestation pregnant women	Interpersonal therapy	Rates of breastfeeding initiation	Professional	NS *P* = 0.74
RCT, *N* = 1222, 1–180 DOL	Werner, Denmark, 2013 [37]	27–20 weeks gestation pregnant women	Hypnosis sessions	Breastfeeding duration: 0–4 weeks 1–4 months	Self-report	NS *P* = 0.43 *P* = 0.95
RCT, *N* = 456, 1–180 DOL	Sikander, Pakistan, 2014 [42]	Third trimester pregnant women	Cognitive-behavioural	EBF 6 months	Professional.	Adjusted hazard ratio 0.40; 95% CI (0.27, 0.60); *P* < 0.001
RCT, *N* = 99, 1–90 DOL	Kao, USA, 2015 [38]	20–35 weeks gestation pregnant women	Group interpersonal psychotherapy	Any breastfeeding until three months postpartum	Professional.	54 days (32.5) vs 21 days (31.6); *P* = 0.013
RCT, *N* = 60	Procelli, USA, 2005 [53]	Just delivered mothers	Music therapy	Positive emotional response on BF efficacy	Professional	*P* = 0.045
RCT, *N* = 50, 1 DOL	Sreekumar, India, 2018 [41]	Third trimester pregnant women	Cognitive-behavioural	Satisfactory LATCH score.	Professional	Odds ratio 10.29; 95% CI (1.16, 91.43); *P* = 0.015
RCT, *N* = 64, 1–98 DOL	Mohd Shukri, Malaysia, 2019 [52]	Third trimester pregnant women	Relaxation therapy	Breast milk intake DOL 84 Hindmilk cortisol DOL 14	Professional	mean difference 227 g/d; 95% CI (430, 24); *P* = 0.033 mean difference − 44.5%; 95% CI (−76.1, −12.9); *P* = 0.007
Cluster-RCT, *N* = 71	Shariat, Iran, 2017 [39]	Second or third trimester pregnant women	Group interpersonal psychotherapy	Any breastfeeding until 12 months after birth	Professional	*P* = 0.028
Not randomised, *N* = 38, 1–42 DOL	O’Connor, USA, 1998 [48]	Mothers who were ≤ 2 days post-partum	Relaxation therapy	Breast milk secretory IgA.	Professional	NS *P* = 0.78
Quasi-experimental, *N* = 220, 1–480 DOL	Patel, India, 2013 [45]	Primipara mothers within 2 h of delivery	Manual relaxation	Post-feed weight gain	Professional	*P* = 0.05
Quasi-experimental, *N* = 29, 1–4 DOL	Ak, India, 2015 [51]	Mothers of preterm infants	Music therapy	Breast milk volume.	Professional	7.12 mL (1.57) vs 6.68 mL (1.37); mean difference 0.44; 95% CI (0.05, 0.82); *P* = 0.033
Pre-post assessment, *N* = 50	Mohammadpour, Iran, 2018 [44]	Mothers of preterm infants	Manual relaxation	Breast milk yield	Self-report	*P* < 0.05
Pre-post assessment, *N* = 262, 1–730 DOL	Mitchell, Australia, 2018 [55]	Mothers who were ≤ 24 months post-partum	Compassion focused relaxation therapy	Maternal Breastfeeding Satisfaction Scale.	Self-report	6.14 (1.43) vs 6.46 (0.97); *P* < 0.001
Within-subject study, *N* = 20, 01730 DOL	Yu, China, 2019 [47]	Mothers who were ≤ 24 months post-partum	Relaxation therapy	Breast milk ejection	Self-report	n/a
Case series, *N* = 3	Tipping, UK, 2000 [46]	Mothers having a complicated delivery	Manual relaxation	Breastfeeding initiation	Professional	n/a
Case series, *N* = 3, 1–365 DOL	Cowley, Canada, 2005 [47]	Postpartum tetraplegic women	Relaxation therapy	Breast milk ejection.	Self-report	n/a
Case series, *N* = 11, 1–8 DOL	Hauck, Australia, 2008 [43]	Breastfeeding women	Relaxation therapy	Perception of breastfeeding success.	Self-report	n/a

*CI* confidence interval, DOL days of life, *EBF* exclusive breastfeeding, *NS* not significant, *n/a* does not apply, *OR* odds ratio, *RCT* randomized controlled trial, *S.D.* standard deviation

**Table 3:** Interventions’ description and study limitations

Publication, study design	Mothers’ mental illness	Intervention	Type of guidance	Comparison groups Extra attention	Study limitations
Cognitive behavioural therapy (CBT)					
Sikander 2014, [42]RCT	no	Seven sessions of CBT were carried out, with women beginning from pregnancy up until six months postpartum. This intervention doubled the rate of exclusive breastfeeding at six months of life	Psycho-educational sessions integrated into the routine work of non specialist heath workers and delivered to all women in their catchment areas	Mothers in the intervention group received 7 sessions of CBT, whereas the control group received an equal number of routine sessions.	This research revealed that the community health workers’ counselling had been too authoritarian, The authors acknowledge that further work is needed to refine the intervention so that it does not undermine the confidence of many women, and it can simultaneously address behaviour change.
Sreekumar 2018, [41]RCT	no	A single session of CBT in the third trimester: an intensive counselling session was carried out with the use of illustrations, along with detailed information regarding the usefulness of early initiation of breastfeeding, the right technique of feeding, and problems.	Specialist lactation counsellors	This study tested the effectiveness of a cognitive approach compared to routine counselling in the third trimester. By simple randomization, 26 mothers underwent cognitive counselling and 24 mothers underwent routine counselling.	Small sample size and the lack of follow-ups with the babies during the first 6 months. An improved LATCH score implies that the mother will have a more successful breastfeeding. However, more studies are needed to confirm the long-term benefits of a single session of CBT.
Interpersonal psychotherapy (IPT)					
Spinelli 2013, [36] RCT	yes	This study examined the comparative effectiveness of IPT and a parenting education program (PEP). Each participant received twelve weeks of an intervention. The PEP includes individual 45-min weekly lectures on pregnancy, postpartum, breastfeeding, and infant development.	A psychiatrist or a social worker.	Although breastfeeding education was not mandatory in the IPT group, a majority of IPT therapists provided breastfeeding encouragement. 83% of the IPT participants and 100% of the PEP participants received this breastfeeding intervention.	Breastfeeding was not defined as exclusive. In addition, the sample was small, and counselling was less than 100%. Given that this was a depression intervention study, the desire for treatment may have biased the outcomes. A benefit of this study is that prospective data on breastfeeding were not subject to recall bias.
Kao 2015, [38] RCT	yes	Four 60-min group sessions over a 4-week period and a 50-min individual booster session after delivery. The sessions provided a review of the symptoms of PPD, addressed stress management skills, identified conflicts around childbirth and techniques for resolving them.	Group leaders were two nurses who received two hours of training and supervision in the delivery of the intervention.	Women were randomized to group sessions plus standard antenatal care or to standard antenatal care alone. Standard antenatal care included no groups for mental health issues, but offered classes on breastfeeding, infant safety, and parenting.	Duration of breastfeeding was only assessed in the three months postpartum period. Variables such as improved self-care and self-efficacy in the postpartum period, were not measured and therefore could not be examined. These findings may not generalize to women at low risk for PPD.
Shariat 2017, [39] Cluster-RCT	no	Pregnant women in the intervention group took part in three supportive psychotherapy group sessions. The mothers were allotted to ask questions at the end of each session. They were also given an instructional package associated with mother-infant attachment behaviors aside from the group sessions.	Clinical psychologist	Women visiting a maternity clinic were randomly assigned to intervention and control groups. The control group was only provided with the routine pregnancy care.	Breastfeeding persistence in the intervention group increased significantly compared to the control group. This increase could be due to the influence of supportive psychotherapy, instructions, interactions, visualization, or more communication with the fetus in the intervention group.
Self-hypnosis					
Werner 2013, [37] RCT	no	The intervention group attended three 1-h classes on self-hypnosis held over three consecutive weeks with additional audio-recordings to ease childbirth.	Women have been trained to guide themselves through a hypnotic procedure by two midwives trained in hypnosis.	A relaxation group receiving lessons in various relaxation methods and mindfulness, and a group receiving only the usual antenatal care were compared with the intervention group.	The intensity of the intervention may have been too limited, which may have reduced the effect. The generalizability of the results of this trial may be limited as the participants scored high on wellbeing and were well-educated.
Compassion-focused relaxation therapy					
Mitchell 2018, [55] Pre-post assessment	no	Online resources comprised two videos and a tip sheet. The first video and the tip sheet explained the concept of self-compassion during the transition to motherhood, and the second video was a guided self-compassion visualisation exercise. Participants could access the videos as many times as they wished during the course of the study.	The online package included practicing self-compassion visualisation exercises.	There was no control group. This longitudinal study used a within-group repeated-measures design (pre-intervention, 1-month post-intervention).	Given the brevity of the intervention and short-duration follow-up, changes from pre- to post-intervention must be interpreted with caution.
Manual relaxation					
Patel 2013, [45] Quasi-experimental	no	The first back massage was given within two hours of delivery and continued for four times a day at regular intervals for three days. After proper exposure and comfortable position, gentle pressure with both thumbs was given on the back, in circulatory motion. This was applied for 15 min at each sitting.	Orientation about the protocol was given by nursing staff taking care of the postnatal wards. Acupressure, also called shiatsu, is a simple, and friendly method of back massage.	The study was conducted over a period of 16 months. Group A was the study group of subjects to whom back massage was given and group B, the control group of subjects to whom routine care was provided.	Other studies have shown similar effects, though the site of massage/acupressure was different. This study assessed the effect of back massage on lactation indirectly.
Mohammadpour 2018, [44] Pre-post assessment	no	In the reflexology group, massage was done in six sessions. Each session lasted about one hour.	The mother first was placed in a comfortable position. Subsequently, the researcher applied continuous pressure to feet and kidneys.	Only routine interventions were performed in the control group, including regular breastfeeding training.	The authors comment that they cannot be sure about the level of interest of the participants when performing the intervention. In addition, participants may have used other milk-enhancing methods.
Environmental sensory stimulations					
Procelli 2005, [53] RCT	no	New mothers who intend to breastfeed received music therapy and relaxation. A minimum duration of 10 min of music therapy was required for the study. When the mother verbalized her readiness to breastfeed the music ceased and observation began.	Live music was sung by the researcher and played on a classical guitar. Mothers were asked their music preference according to favourite songs. During the music therapy intervention, subjects engaged in relaxation techniques and counselling techniques.	Two-group study, experiment and control, post-test design with random assignment of subjects. The intervention was music therapy paired with relaxation techniques.	Prior research indicated that performing a routine before each breastfeeding experience can enhance her milk production. Music therapy allowed the mother to have at least ten minutes of time exclusively for her and her baby. In this case, music therapy itself cannot be disentangled from setting a routine to prepare the mother.
Keith 2012, [50] RCT	no	The three experimental groups received mp3 players with a recording of approximately 12 min in duration. All recordings consisted of a spoken progressive muscle relaxation protocol, followed by a guided imagery protocol.	Each mother was asked to listen to the mp3 player on headphones as often as possible while using the breast pump	Mothers who chose to participate were randomized into 3 experimental groups and the control group. Each group received standard medical, nursing, lactation education, and support in initiating and maintaining breast milk production. Generally, mothers were encouraged to pump 8 times daily for about 10 min.	The results suggest that mothers who heard the verbal-only protocol produced more milk than those who heard both verbal and music. This brings up one question: did the simultaneous presentation of two audio stimuli represent a distraction for the participants?
Ak 2015, [51] Quasi-experimental	no	Music therapy was given to mothers of preterm babies when they went to the NICU to express breast milk in a randomized manner, without an accompanying verbal protocol, when they were seated comfortably in a quiet room, at a comfortable level of volume of their choice.	Study subjects were trained to use the breast milk pump. A total of 30 min rendition of the raga Malkauns and Yaman played on the flute was used for music therapy. Mothers heard the music with earphones.	Cross-over trial. Each subject was assessed for 4 sessions on Music Therapy and 4 sessions on No Music Therapy.	In the absence of any literature providing the yield of breast milk with and without music therapy, a sample size of 30 was fixed. Salivary cortisol was used as an indirect measure of stress. As it was a nonparametric data, Wilcoxon rank test was applied.
Auditory-mediated exercises					
Feher 1989, [54] RCT	no	Mothers in the treatment group received a cassette tape. The tape consisted of a progressive relaxation exercise followed by a guided imagery section. Progressive relaxation involves alternately tensing and relaxing muscle groups while taking deep breaths. The guided images included descriptions of pleasant surroundings, and the baby’s warm skin against the mother.	The treatment tape was made by the senior author, who was a man. It is possible that a female voice could increase the effectiveness of the tape.	After the initial interview, the women were randomly assigned to the intervention or comparison groups. All mothers received routine supportive care from the nursing staff, including verbal and written instructions concerning the use of the electric breast pump.	More clear-cut differences in creamatocrits might have been observed in this study if the time of day of sample collection had been standardized. Compared with the mothers in the intervention group, the group of control mothers were more likely to be primiparous, and were less likely to be affluent.
Vidas 2011, [40] RCT	no	Mothers from the experimental group were taught autogenic training for 12 weeks. Autogenic training is a suggestion-supportive psychotherapy technique. Experienced physical changes lead to psychological relaxation.	Mothers in the intervention group were learning autogenic training with the trainer. Every two weeks they were practicing a new exercise. The six basic exercises of autogenic training were taught in small groups to 10 members.	Mothers from experimental and control groups were advised for successful breastfeeding up to six months of age. Both groups were equally satisfied with the health care in order to protect breastfeeding.	Despite the high satisfaction with counselling, mothers were more satisfied when they used autogenic training. According to the authors, this could mean that autogenic training plays a critical role in their success at breastfeeding.
Mohd Shukri 2019, [52] RCT	no	After each home visit, mothers in the intervention group were asked to listen to the therapy daily while breastfeeding for at least 2 wk. They were also encouraged to listen beyond 2 wk. as frequently as they found useful throughout the trial and to record in a diary when it was used; the duration of the intervention was 12 wk.	The relaxation therapy audiorecording was a modified audio-guided imagery protocol designed for breastfeeding mothers.	Mothers in both groups received standard breastfeeding support during the trial (standard breastfeeding education materials such as a breastfeeding guidance booklet, as well as a list providing contact details of health practitioners in health clinics, breastfeeding support groups, and lactation counsellors in their area).	First, no adjustment of sample size was performed for the primary outcomes. Thus, the possibility of a type 1 error should be considered when interpreting the findings. Second, due to the nature of the therapy tool, it was not possible to blind mothers or researchers to the intervention.
O’Connor 1998, [48] Quasi-experimental	no	Women in group 1 were encouraged to practice the relaxation once or twice a day for two weeks, and a second visit was made to all mothers with repeated breast milk collections; women who were still breast-feeding at 6 weeks after study end had a final breast milk sample collected. Women in group 2 had a conversation with similar breast milk sample collection. And women in group 3 had 1 breast milk sample collected.	Participants varied in their mental imagery preferences. Some described excellent visual imagery, whereas others preferred auditory or olfactory imagery.	Study groups: relaxation intervention, attention control, and no intervention.	This study explores relaxation success: recent mothers rated from very good to poor at relaxation. Inasmuch as the authors attempted to use the same relaxation coaching for each subject, they did not allow sufficiently for individual differences.
Yu 2019, [47] Quasi-experimental, within-subject study	no	Primiparous mothers attended relaxation meditation tape, music tape, relaxation lighting, or combined relaxations sessions. The tape was recorded by a certified yoga therapist. Participants could choose their preferred music to enhance stress reduction. Participants could choose either the orange light (“Relax” setting) or the blue light (“Energize” setting) to meet their preference.	To control for circadian rhythm, all sessions were performed in the afternoon between 2:00 PM and 4:00 PM. The duration of each treatment was 10 min, with additional 10-min pre- and post- test measurements.	A within-subject study on mothers allocated in randomised order to different relaxation sessions and control sessions, with a washout period of 1–3 days between sessions.	Only 20 participants from one community were enrolled, so the population may not be representative. A potential disadvantage of the within-subjects design is that there might be “carryover effects” of one intervention on the next.
Case series					
Cowley 2005, [49] Case series, Pharmacotherapy and active mental imaging relaxation techniques	no	After learning how to recognize the signs of milk ejection, paraplegic mothers of five neonates used a process of relaxation and imaging to mentally elicit a let-down reflex. In the immediate postnatal period, one mother used an oxytocin nasal spray to enhance let-down.	Mothers watch their infants’ suckling behaviour, and as soon as their milk flow is starting to decrease, they begin the mental process to elicit a second let-down, through a series of thoughts that involved images of nurturing their infants.	There is no control group. However, the initial failure of the mother in case 1 to thrive her twins without relaxation techniques suggests that imaging techniques foster breastfeeding.	In this series, no measurements were made with which to correlate the mothers’ perceptions of let-down with increased intramammillary pressure. Therefore, it is possible that these mothers were able to achieve let-down without active mental relaxation techniques
Tipping 2000, [46] Case series, Manual relaxation	no	In all cases, a reflexologist was asked to see a mother who was having difficulty with the initiation of lactation. At this time a reflexology treatment to activate the whole body of the mother is offered.	Teaching reflexology techniques to parents to empower them to have control over at least one part of their baby’s care.	Reflexology cannot take all the credit for the increase in lactation. There is no control group. Many women report self-esteem or confidence increase when supported in comprehensive ways.	The numbers involved were too small to substantiate that reflexology could reduce the particular stress involved with lactating mothers of a neonate.
Hauck 2008, [43] Case series, Environmental sensory stimulations	no	A relaxation room has soft earthy colours and provides a chaise where a mother can lie and relax. The relaxing environment includes a fish tank, music and aromatherapy.	Information about the relaxation room and its aim to promote relaxation is provided during hospital tours and antenatal classes. Midwives encourage use of the room for relaxation for breastfeeding women, women in early labour, and anxious women.	A qualitative exploratory design was employed to obtain a rich description of the experience of using the relaxation room. No control group.	The researchers are aware that a Snoezelen room is just one example that may be considered to assist breastfeeding mothers to achieve relaxation, and anticipate that the description of these findings will enable the reader to determine the transferability of the findings to their own context.

*BF* breastfeeding, *CBT* cognitive behavioural therapy, *CI* confidence interval, *DOL* days of life, *EBF* exclusive breastfeediing, *GA* gestational age, *LATCH* acronym of Latching of infant onto the breast, Amount of audible swallowing, Type of nipple, Comfort of mother, Help needed by mother to hold baby to breast.; *NS* not significant, *n/a* does not apply, *OR* odds ratio, *PPD* postpartum depression, *RCT* randomized controlled trial, *S.D.* standard deviation, *wk.* weeks

### Cognitive behaviour therapy programs

Cognitive behavioral therapy (CBT) refers to a group of interventions that combine cognitive and emotion-focused techniques, with the objective of replacing unreal and negative beliefs with more precise and positive thoughts. This type of therapy was used in a study in rural Pakistan, where various sessions of CBT were carried out by trained health workersThis intervention doubled the rate of exclusive breastfeeding at six months of life [42]. Similarly, a project in India reports that a single session of CBT was effective in significantly improving how well the infant latches onto the breast in the immediate neonatal period [41].

### Interpersonal psychotherapy

Interpersonal psychotherapy (IPT) is based on attachment theory [56, 57]. Our review identified three studies investigating the effects of IPT on breastfeeding outcomes. One study from the New York State Psychiatric Institute, examined the comparative effectiveness of IPT and a parenting education program (PEP), in promoting breastfeeding duration among pregnant women who met DSM-IV criteria for a major depressive episode [36]. Irrespective of the fact that IPT or PEP was administered, 76% of the women were breastfeeding at the fourth postpartum week. These researchers point out that their breastfeeding rates post-intervention exceed consistent findings in the literature. The other two studies assessed the effectiveness of IPT group intervention, with regards to breastfeeding success. One of them, the ROSE program consisted of four group sessions for 3–5 pregnant women at high risk of perinatal depression [38]. Though women in the ROSE program had similar breastfeeding initiation rates, as compared to women on standard care, they maintained breastfeeding for longer. Finally, in a study on women visiting a maternity clinic in Tehran, women in the intervention group took part in three supportive psychotherapy sessions and, thereafter, the rate of breastfeeding increased significantly.

### Hypnosis

Hypnosis has previously been found to provide pregnant women with skills to successfully manage labour [58–60]. A Danish RCT compared the breastfeeding outcomes of three groups of nulliparous healthy women: a self-hypnosis group, a mindfulness relaxation group, and a usual-care group [37]. Concerning self-hypnosis, there was no difference in establishing breastfeeding or breastfeeding duration between the intervention and the two control groups.

### Relaxation techniques

Various relaxation methods share a long history of clinical practice and research. Such therapies have the potential to both add quality to breastfeeding matters, and to alleviate stress during the postpartum period [61, 62]. We analysed 13 studies that examined the effect of these interventions on breastfeeding practice. One study was devoted to compassion-focused relaxation therapy [55], and another isolated study combined pharmacotherapy with active mental imaging relaxation techniques [49]. Three papers explore manual relaxation as an integrative psychotherapy approach [44–46]. Four studies address the influence of various environmental sensory stimulations on milk production [43, 50, 51, 53]. Finally, auditory-mediated exercises that guide the body to relax and control breathing, thus alleviating stress and promoting breastfeeding were analysed in five research papers [40, 47, 48, 52, 54].

Compassion-focused therapy is a system of psychotherapy designed to help individuals being kind to one-self and being mindful, in order to reduce high levels of self-criticism [63]. An Australian study sought to evaluate the potential utility of a package of online resources designed to improve self-compassion for mothers within 24 months postpartum [55]. Mean total scores for the Maternal Breastfeeding Evaluation Scale [64] indicated improvement from pre- to post-intervention.

A case series illustrates that women with tetraplegia can sustain breastfeeding for extended periods [49]. Active mental imaging and relaxation efforts on the part of the mother were required to facilitate let-down in two cases. Later on, one of these cases needed a nasal spray of oxytocin after receiving oxycodone for pain relief. The third tetraplegic mother was successful in her attempts to breastfeed without additional relaxation or drug therapy.

Three different manual relaxation papers have been evaluated [44–46]. A case series [46], and a clinical trial using a pretest–posttest design has examined reflexology [44]. Reflexology is a treatment in which pressing some areas (hands, feet or ears) can cause deep relaxation and result in the secretion of hormones. In a case series of three mothers after an obstructed labour, reflexology three times a week was shown to increase milk secretion. Similarly, a trial showed that the increase in milk volume from day 1 to day 5 after the reflexology intervention was significantly higher in the intervention group, compared to the control group. When it comes to back massage, a quasi-experimental study was conducted to assess the effectiveness of this relaxation technique in post-feeds weight gain, among primiparous mothers of full-term neonates [45]. The intervention started immediately after birth. It was found that the mean frequency of micturition and post-feed weight gain was higher among infants in the study group.

Four studies investigated the association between specific environmental sensory stimulations and breastfeeding success [43, 50, 51, 53]. In one study, participants entered the Snoezelen room with an unsettled baby and breastfeeding issues aggravated by maternal tiredness [43]. The main features of the room are the wheel projection that slowly rotates to display patterns on the wall, a tropical fish tank, music and aromatherapy. Eleven women shared their experience using the Snoezelen room during the early stages of their breastfeeding, 80% of them achieved exclusive breastfeeding and all of them indicated they would recommend its use to other breastfeeding mothers.

Two studies on music therapy involved mothers of preterm infants. In one of them [50], the control group received only the standard support, whilst the other three experimental groups received mp3 players with a recording of a spoken progressive muscle relaxation protocol, accompanied or not by selections of lullabies for guitar or by a series of images of the mother’s infant. In particular, the intervention that included a slideshow of images of the mother’s child improved breast milk quantity more than the others. The other study was carried out on mothers of preterm babies when they went to the NICU to express breast milk [51]. Mothers’ salivary cortisol level showed a significant decrease, and mean volume of expressed breast milk showed a significant increase after music therapy. Another study recruited 60 women in-hospital who had just delivered their first child, and had the intention to breastfeed. The independent variable was music therapy paired with relaxation techniques. Mothers who received a longer music therapy session ended up breastfeeding for a longer amount of time and their perceptions of their experience were more positive [53].

Finally, five studies examined the effect of auditory-mediated mind guidance on breastfeeding practice [40, 47, 48, 52, 54]. The first results on promoting breastfeeding through these techniques dates back 30 years. Mothers in the intervention group were instructed to listen every day to a 20-min audio that consisted of a progressive relaxation exercise followed by a guided imagery section. Approximately one week after enrolment, the mean volume of expressed milk in the intervention group was 63% greater than the mean volume in the no intervention group [54]. Another study sought to clarify the effect of stress on breast milk humoral immunomodulation. A convenience sample of healthy breastfeeding women was recruited within 48 h of delivery. The women were assigned to one of the following three study groups: relaxation intervention (group 1), attention control (group 2), and no intervention (group 3). Milk samples were tested for sIgA five times, until six weeks postpartum. The sIgA levels in breast milk samples from women in each of the intervention groups were not significantly different. However, this paper presents the intriguing results that sIgA levels in group 1 varied with relaxation success: the women who rated very good and poor at relaxation, had levels of 0.30 and 0.67 g/L, respectively. This is opposite to the concept that cortisol may suppress the immunoglobulin production by plasma cells in the breast [48]. The unique research on autogenic relaxation was a randomized prospective study that analysed data of breastfed infants from the second month of age until the age of six months [41]. In the experimental group, 47 out of 50 mothers (94%) fully breastfed their child during the first six months of life, while those in the control group were 17 (34%) [41]. In 2019, two groups of researchers unveiled details of how relaxation techniques affect breastfeeding outcomes. A Chinese within-subject study on 20 primiparous mothers who were breastfeeding compared basic physiological parameters from five different approaches to relaxation: meditation tape (RM), music tape (M), relaxation lighting (L), combined RM + L, combined M + L, and control session without intervention [47]. Heart rate (HR), blood pressure (BP), fingertip temperature, and the let-down breastfeeding reflex were assessed before and after each session. RM resulted in the greatest change in BP and HR, and four participants experienced milk let-down (ejection reflex) during the session. This was not found for the remaining four treatment or control groups.

The last study recruited healthy first-time mothers during their third trimester of gestation [52]. At two weeks postpartum, mothers and exclusively breastfed full-term infants were randomly assigned to receive relaxation therapy, or to the control group. Mothers in the relaxation group received a modified audio-guided imagery protocol designed for breastfeeding mothers to listen to while breastfeeding, during each home visit. They were also encouraged to listen to it as frequently as they found useful, throughout the 12 weeks trial. Analysis with a linear model showed that infants from the relaxation group had a mean milk intake at 12 weeks that was 226.5 g/d higher than the intake of infants in the control group.

## Discussion

### Summary of evidence

The purpose of this qualitative review is to examine the relationship between perinatal psychological interventions and breastfeeding outcomes. Out of the 20 studies included, three studies reported no relationship between these variables: relaxation techniques do not increase milk sIgA levels, and a brief course of self-hypnosis, or individual sessions of interpersonal psychotherapy have no effect on breastfeeding duration. Five studies with small sample sizes (*n* < 30) may have lacked sufficient power. Importantly, all studies on mothers of preterm infants provide strong evidence in favour of the potential that simple relaxation interventions have for improving the success of milk expression. These cheap techniques may help mothers who are especially stressed during the postpartum period and at a high risk of breastfeeding failure.

According to the levels of evidence developed by the Centre for Evidence Based Medicine for treatment [65], all psychotherapy-based interventions represent level-I or level-II evidence (large RCT trials with clear cut results or small RCT with unclear results, respectively). Based on the quality of evidence to support or oppose these outcomes, psychotherapy appears to be beneficial for these mother-infant dyads, but patient preference should have a substantial role in choosing an appropriate practice.

As for research on relaxation techniques, five studies represented level-I evidence, five level-II evidence, and three level-IV evidence. Based on the quality of evidence and consistent findings, clinicians should recommend relaxation techniques in support of breastfeeding but should remain alert to new information.

### Discussion of findings

Research on psychological interventions intended to support breastfeeding practices has largely been neglected over the years. As far as we know, only three very recent reviews have focused on the effects of behaviour-change or stress-releasing techniques on breastfeeding success, or breast milk composition [66–68]. A 2018 critical analysis has assessed the effectiveness of body and mind stress-releasing techniques on the breastfeeding success of mothers of healthy neonates [66]; nine studies were included in this analysis. Researchers conclude that cognitive-behavioural counselling seems effective in improving breastfeeding initiation and duration, that manual relaxation techniques seem effective in promoting breastfeeding initiation, and that mind guidance seems also effective in promoting breastfeeding duration. A 2018 systematic review [67] has identified four studies investigating the effects of relaxation therapy on breast milk yield and composition. Among them, two RCTs [50, 54] found that relaxation therapy significantly increased milk yield. In terms of milk composition, one RCT [50] reported a significant increase in fat content in the breast milk of mothers in the intervention group. A 2019 meta-analysis [68] aimed to investigate the effectiveness of socio-psychological interventions in promoting breastfeeding initiation, duration or exclusivity. Reviewers found 20 studies that employed behavioural change techniques, with a majority of interventions falling into the category of Education and Support. After adjusting for publication bias, socio-psychological interventions reviewed did not increase rates of any breastfeeding across the postpartum period.

To the best of our knowledge, our review is the first one to comprise all psychological interventions intended to assist mothers in need of extra support to breastfeed. Psychotherapy and relaxation techniques are necessary to deal with some cases that are not adequately addressed by peer-support programmes or public health initiatives, implemented to create environments to support breastfeeding. Our findings are in line with the effectiveness of previously reported interventions offering consistent psychological support in the *peripartum* period, despite the inherent differences in psychological mechanisms of action or manner of psychological well-being promotion.

Given the proven interaction between the reproductive and stress systems [69, 70], there is no doubt of the impact of stress-releasing techniques on lactation. However, most interventions included in this review did not theorise specifically on how the interventions were intended to work. This is all part of the majority of successful breastfeeding interventions whose mechanism of action remains unknown [6, 8, 71].

### Limitations

Systematic reviews have increasingly replaced traditional narrative reviews to update the best evidence for the most basic and clinical questions. The major advantage of systematic reviews is that they are based on the findings of comprehensive literature searches in all available resources, avoiding subjective selection bias, while narrative reviews can provide the authors’ experiential perspectives in focused topics.

This narrative review is not focused on a specific question. It is conceived as a descriptive overview of a topic. As concepts can be expressed in different ways, combinations of keywords and subject headings have been used to look at all the evidence. If a study was left out, it was by mere chance and not because of the study’s results. This kind of search strategy has a higher sensitivity, it is used to minimize bias, but often lowers the precision (relevance or accuracy) of the results. Additionally, there are several search tactics for which there is no consensus. Variation in practice around such issues as limits, searching for observational studies, for outcomes and comparators, persists over an extended period of time [72, 73].

While the small number of studies included in this review reflects the current status of this novel approach to breastfeeding support, it also reduces the strength of the respective recommendations, due to the difficulty in obtaining pooled data. Some studies had a small sample size, which may not provide sufficient power to detect differences in outcomes. Despite employing broad eligibility criteria to include interventions, inclusion of published studies may have introduced some bias towards studies with positive findings. The lack of a clear breastfeeding definition decreased the comparability of research results. Current definitions for breastfeeding have been questioned. Clinicians and statisticians need accurate definitions for breastfeeding. Many studies provided varying cut-offs and most studies evaluated infant-feeding methods at various times throughout the postpartum period; heterogeneity in measurements limits their comparability. The majority of the reviewed studies do not distinguish between exclusive and partially breastfed infants. We elected to accept all definitions of “exclusive breastfeeding” as provided by the different study authors. Another limitation was that several studies incorporated self-report measures, hence there may have been underreporting of the use of nonhuman milk because previous research suggests that preventive infant-feeding practices tend to be overestimated by self-report measures. In addition, with lengthy time-periods involved, retrospective studies may have some degree of recall bias.

This review acknowledges that there is a particular instance where mixed research methods may contribute in assembling evidence from quantitative studies and in-depth analyses of few patients.

Although the same subjects were not expected to be included, multi-centre studies have been excluded because a cluster effect might occur; in exchange, there is a risk of losing larger studies.

Finally, generalizability of results may have also been limited because only half of studies controlled for potential confounding variables such as maternal age, education or family income in their multivariate analyses.

## Conclusions

The studies included in this review show that psychotherapy as well as stress-releasing interventions, seem to support breastfeeding success. Conversely, this review suggests that self-hypnosis does not lead to improvements in breastfeeding outcomes. Concerning population groups, the strongest evidence was for an effect in increasing milk volume expressed by mothers of preterm infants. Future research is needed to evaluate the impact of psychological interventions on breastfeeding duration, to enable high-quality evidence to be implemented into practice.

## Data Availability

Not applicable.

## Abbreviations

BP: Blood pressure
CBT: Cognitive behavioral therapy
HR: Heart rate
IPT: Interpersonal psychotherapy
NICU: Neonatal intensive care unit
PED: Parental education program
PMD: Perinatal mood disorders
RCT: Randomized controlled trial

## References

[1] Stuebe AM (2015). Does breastfeeding prevent the metabolic syndrome, or does the metabolic syndrome prevent breastfeeding?. Semin Perinatol..

[2] Kendall-Tackett K (2007). A new paradigm for depression in new mothers: the central role of inflammation and how breastfeeding and anti-inflammatory treatments protect maternal mental health. Int Breastfeed J..

[3] Moore ER, Bergman N, Anderson GC, Medley N (2016). Early skin-to-skin contact for mothers and their healthy newborn infants. Cochrane Database Syst Rev..

[4] Becker GE, McCormick FM, Renfrew MJ (2008). Methods of milk expression for lactating women. Cochrane Database Syst Rev..

[5] Centers for Disease Control and Prevention. Strategies to Prevent Obesity and Other Chronic Diseases: The CDC Guide to Strategies to Support Breastfeeding Mothers and Babies. Atlanta. Department of Health and Human Services. 2013; 10.3945/an.114.005900. (last time accessed 11th October, 2020). 10.3945/an.114.005900 PMC4013182 24829476

[6] Fairbank L, O'Meara S, Renfrew MJ, Woolridge M, Sowden AJ, Lister-Sharp D (2000). A systematic review to evaluate the effectiveness of interventions to promote the initiation of breastfeeding. Health Technol Assess..

[7] Balogun OO, O'Sullivan EJ, McFadden A, Ota E, Gavine A, Garner CD, Renfrew MJ, MacGillivray S (2016). Interventions for promoting the initiation of breastfeeding. Cochrane Database Syst Rev..

[8] Haroon S, Das JK, Salam RA, Imdad A, Bhutta ZA (2013). Breastfeeding promotion interventions and breastfeeding practices: a systematic review. BMC Public Health.

[9] Hamilton JA (1962). Chapter 12, History: In Postpartum Psychiatric Problems.

[10] den Besten-Bertholee D, van der Meer DH, Ter Horst PGJ (2019). Quality of lactation studies investigating antidepressants. Breastfeed Med..

[11] Charlton RA, Jordan S, Pierini A, Garne E, Neville AJ, Hansen AV (2015). SSRI use before, during and after pregnancy: a population-based study in 6 European regions. BJOG..

[12] Molyneaux E, Telesia LA, Henshaw C, Boath E, Bradley E, Howard LM (2018). Antidepressants for preventing postnatal depression. Cochrane Database Syst Rev..

[13] Jordan S, Davies GI, Thayer DS, Tucker D, Humphreys I (2019). Antidepressant prescriptions, discontinuation, depression and perinatal outcomes, including breastfeeding: A population cohort analysis. PLoS one..

[14] Grigoriadis S, VonderPorten EH, Mamisashvili L, Tomlinson G, Dennis CL, Koren G (2013). The impact of maternal depression during pregnancy on perinatal outcomes: a systematic review and meta-analysis. J Clin Psychiatry..

[15] Cooper PJ, Murray L, Stein A (1999). Psychosocial factors associated with the early termination of breast-feeding. J Psychosom Res..

[16] Misri S, Sinclair DA, Kuan AJ (1997). Breast-feeding and postpartum depression: is there a relationship?. Can J Psychiatry..

[17] Galler JR, Harrison RH, Biggs MA, Ramsey F, Forde V (1999). Maternal moods predict breastfeeding in Barbados. J Dev Behav Pediatr..

[18] Papinczak TA, Turner CT (2000). An analysis of personal and social factors influencing initiation and duration of breastfeeding in a large Queensland maternity hospital. Breastfeed Rev..

[19] Henderson JJ, Evans SF, Straton JA, Priest SR, Hagan R (2003). Impact of postnatal depression on breastfeeding duration. Birth..

[20] Righetti-Veltema M, Conne-Perréard E, Bousquet A, Manzano J (2002). Postpartum depression and mother-infant relationship at 3 months old. J Affect Disord..

[21] Taveras EM, Capra AM, Braveman PA, Jensvold NG, Escobar GJ, Lieu TA (2003). Clinician support and psychosocial risk factors associated with breastfeeding discontinuation. Pediatrics..

[22] McLearn KT, Minkovitz CS, Strobino DM, Marks E, Hou W (2006). Maternal depressive symptoms at 2 to 4 months post partum and early parenting practices. Arch Pediatr Adolesc Med..

[23] Slomian J, Honvo G, Emonts P, Reginster JY, Bruyère O (2019). Consequences of maternal postpartum depression: A systematic review of maternal and infant outcomes. Womens Health (Lond).

[24] Shulpis K, Iakovou K (2019). Replacement of breastfeeding with medical food for the treatment of galactosemia and phenylketonuria: Maternal stress. J Pediatr Endocrinol Metab..

[25] Maes M, Lin A-H, Ombelet W, Stevens K, Kenis G, Dejongh R (2000). Immune activation in the early puerperium is related to postpartum anxiety and depression symptoms. Psychoneuroendocrinology..

[26] Schiepers OJ, Wichers MC, Maes M (2005). Cytokines and major depression. Prog Neuropsychopharmacol Biol Psychiatry..

[27] Heinrichs M, Meinlschmidt G, Neumann I, Wagner S, Kirschbaum C, Ehlert U, Hellhammer DH (2001). Effects of suckling on hypothalamic-pituitary-adrenal axis responses to psychosocial stress in postpartum lactating women. J Clin Endocrinol Metab..

[28] Mezzacappa ES, Katlin ES (2002). Breast-feeding is associated with reduced perceived stress and negative mood in mothers. Health Psychol..

[29] Groer MW (2005). Differences between exclusive breastfeeders, formula-feeders, and controls: A study of stress, mood, and endocrine variables. Biol Res Nurs..

[30] Groer MW, Davis MW (2006). Cytokines, infections, stress, and dysphoric moods in breastfeeders and formula feeders. J Obstet Gynecol Neonatal Nurs..

[31] Newton M, Newton NR (1948). The let down reflex in human lactation. J Pediatr..

[32] Ueda T, Yokoyama Y, Irahara M, Aono T (1994). Influence of psychological stress on suckling induced pulsatile oxytocin release. Obstet Gynecol..

[33] Ballard M, Montgomery P (2017). Risk of bias in overviews of reviews: a scoping review of methodological guidance and four-item checklist. Res Synth Methods..

[34] Rana KF, Saeed A, Shamim SA, Tariq MA, Malik BH (2019). The association between hypertensive disorders of pregnancy and peripartum cardiomyopathy. Cureus..

[35] AsPredicted Protocol 'Perinatal psychological interventions to promote breastfeeding'. (#40903). Publicly available at: https://aspredicted.org/dj5t7.pdf. Accessed 1 Nov 2020.

[36] Spinelli MG, Endicott J, Goetz RR (2013). Increased breastfeeding rates in black women after a treatment intervention. Breastfeed Med..

[37] Werner A, Uldbjerg N, Zachariae R, Nohr EA (2013). Effect of self-hypnosis on duration of labor and maternal and neonatal outcomes: a randomized controlled trial. Acta Obstet Gynecol Scand.

[38] Kao JC, Johnson JE, Todorova R, Zlotnick C (2015). The positive effect of a group intervention to reduce postpartum depression on breastfeeding outcomes in low-income women. Int J Group Psychother..

[39] Shariat M, Abedinia N (2017). The effect of psychological intervention on mother-infant bonding and breastfeeding. Iran J Neonatol..

[40] Vidas M, Folnegović-Smalc V, Catipović M, Kisić M (2011). The application of autogenic training in counseling center for mother and child in order to promote breastfeeding. Coll Antropol..

[41] Sreekumar K, D'Lima A, Silveira MP, Gaonkar R (2018). Cognitive Breastfeeding Counseling: A Single Session Helps Improve LATCH Score. J Perinat Educ..

[42] Sikander S, Maselko J, Zafar S, Haq Z, Ahmad I, Ahmad M, Hafeez A, Rahman A (2015). Cognitive-behavioral counseling for exclusive breastfeeding in rural pediatrics: a cluster RCT. Pediatrics.

[43] Hauck YL, Summers L, White E, Jones C (2008). A qualitative study of Western Australian women’s perceptions of using a Snoezelen room for breastfeeding during their postpartum hospital stay. Int Breastfeed J..

[44] Mohammadpour A, Valiani M, Sadeghnia A, Talakoub S (2018). Investigating the effect of reflexology on the breast milk volume of preterm infants' mothers. Iran J Nursing Midwifery Res..

[45] Patel U, Gedam DS, Verma M (2013). Effect of back Massage on Lactation among Postnatal Mothers. Int J Med Res Rev..

[46] Tipping L, Mackereth PA (2000). A concept analysis: the effect of reflexology on homeostasis to establish and maintain lactation. Complement Ther Nurs Midwifery..

[47] Yu J, Wells J, Wei Z, Fewtrell M (2019). Randomized trial comparing the physiological and psychological effects of different relaxation interventions in Chinese women breastfeeding their healthy term infant. Breastfeed Med..

[48] O'Connor ME, Schmidt W, Carroll-Pankhurst C, Olness KN (1998). Relaxation training and breast milk secretory IgA. Arch Pediatr Adolesc Med..

[49] Cowley KC (2015). Breastfeeding by women with tetraplegia: some evidence for optimism. Spinal Cord..

[50] Keith DR, Weaver BS, Vogel RL (2012). The effect of music-based listening interventions on the volume, fat content, and caloric content of breast milk-produced by mothers of premature and critically ill infants. Adv Neonatal Care..

[51] Ak J, Lakshmanagowda PB, Pradeep GCM, Goturu J (2015). Impact of music therapy on breast milk secretion in mothers of premature newborns. J Clin Diagn Res..

[52] Mohd Shukri NH, Wells J, Eaton S, Mukhtar F, Petelin A, Jenko-Pražnikar Z, Fewtrell M (2019). Randomized controlled trial investigating the effects of a breastfeeding relaxation intervention on maternal psychological state, breast milk outcomes, and infant behavior and growth. Am J Clin Nutr..

[53] Procelli DE (2006). The effects of music therapy and relaxation prior to breastfeeding on the anxiety of new mothers and the behavior state of their infants during feeding [dissertation].

[54] Feher SD, Berger LR, Johnson JD, Wilde JB (1989). Increasing breast milk production for premature infants with a relaxation/imagery audiotape. Pediatrics..

[55] Mitchell AE, Whittingham K, Steindl S, Kirby J (2018). Feasibility and acceptability of a brief online self-compassion intervention for mothers of infants. Arch Womens Ment Health..

[56] O’Hara MW, Stuart S, Gorman LL, Gorman LL, Wenzel A (2000). Efficacy of interpersonal psychotherapy for postpartum depression. Arch Gen Psychiatry..

[57] Grote NK, Swartz HA, Geibel SL, Zuckoff A, Houck PR, Frank E (2009). A randomized controlled trial of culturally relevant, brief interpersonal psychotherapy for perinatal depression. Psychiatr Serv..

[58] Martin AA, Schauble PG, Rai SH, Curry RW (2001). The effects of hypnosis on the labor processes and birth outcomes of pregnant adolescents. J Fam Pract..

[59] Mehl-Madrona LE (2004). Hypnosis to facilitate uncomplicated birth. Am J Clin Hypn..

[60] Cyna AM, Andrew MI, McAuliffe GL (2006). Antenatal self-hypnosis for labour and childbirth: a pilot study. Anaesth Intensive Care..

[61] Hill PD, Aldag JC, Chatterton RT, Zinaman M (2005). Psychological distress and milk volume in lactating mothers. West J Nurs Res..

[62] Jackson P (2010). Complementary and alternative methods of increasing breast milk supply for lactating mothers of infants in the NICU. Neonatal Netw..

[63] Gilbert P (2014). The origins and nature of compassion focused therapy. Br J Clin Psychol..

[64] Leff EW, Jefferis SC, Gagne MP (1994). The development of the maternal breastfeeding evaluation scale. J Hum Lact..

[65] OCEBM Levels of Evidence Working Group. “The Oxford 2011 Levels of Evidence”. Oxford Centre for Evidence-Based Medicine. http://www.cebm.net/index.aspx?o=5653. (last time accessed 11th October, 2020).

[66] Fotiou C, Siahanidou T, Petros V, Vlastarakos PV, Tavoulari EF, Chrousos G (2018). The effect of body and mind stress-releasing techniques on the breastfeeding of full-term babies; a critical analysis of published interventional studies. J Matern Fetal Neonatal Med..

[67] Mohd Shukri NH, Wells JCK, Fewtrell M (2018). The effectiveness of interventions using relaxation therapy to improve breastfeeding outcomes: A systematic review. Matern Child Nutr..

[68] Davie P, Chilcot J, Chang YS, Norton S, Hughes LD, Bick D (2019). Effectiveness of social-psychological interventions at promoting breastfeeding initiation, duration and exclusivity: a systematic review and meta-analysis. Health Psychol Rev..

[69] Jacobs GD, Friedman R (2004). EEG spectral analysis of relaxation techniques. Appl Psychophysiol Biofeedback..

[70] Douglas AJ (2010). Baby love? Oxytocin-dopamine interactions in mother-infant bonding. Endocrinology..

[71] Renfrew MJ, McCormick FM, Wade A, Quinn B, Dowswell T (2012). Support for healthy breastfeeding mothers with healthy term babies. Cochrane Database Syst Rev..

[72] Del Mar C, Glasziou P (2001). Studying systematic reviews. J Fam Pract..

[73] Robinson KA, Dickersin K (2002). Development of a highly sensitive search strategy for the retrieval of reports of controlled trials using PubMed. Int J Epidemiol..

